# Supplementary material for: Did he or didn’t he? Mixed evidence for the continued influence of retracted misinformation on person impressions
Source: PLoS One. 2025 May 7;20(5):e0322045. doi: 10.1371/journal.pone.0322045 (PMC12058158; doi:10.1371/journal.pone.0322045)
Supplement: S1 File — (PDF) [file pone.0322045.s001.pdf]

S1 File. Pilot Testing Coherence-Building Statements

S1 Text. Pilot Testing Coherence-Building Statements for Experiments 1 and 2

Prior to Experiment 1 and 2, we recruited 100 U.S.-based Prolific workers (54 women, 44 men, 1 other, 1 preferred not to say) aged 19–76 (*M* = 37.01, *SD* = 13.94) to pilot-test coherence-building statements. The pilot test took approximately 11 minutes, and participants were paid £2.00 (approximately US$2.50) for their participation. The results of the pilot testing are shown in Table S1.

S2. Pilot Testing Coherence-Building Statement for Experiment 3

Prior to Experiment 3, a separate pilot test was conducted based on two statements generated by the authors. A sample of 100 U.S.-based Prolific workers (50 women, 49 men, 0 other, 1 prefer not to say) aged 20–65 (*M* = 37.18, *SD* = 11.14) was recruited. The pilot test took approximately 2 minutes, and participants were paid £0.45 (approximately US$0.60) for their participation. Results are shown in Table S1.

S1 Table. Candidate Coherence-Building Statements and Descriptive Statistics

Scenarios	Morality (−4 to 4)	Believability (0 to 8)	Imply (1 to 7)	Explain (1 to 7)
**Scenarios related to affair-target (Experiment 1)**				
John and his wife met with a marriage therapist.*	2.14	7.16	3.48	4.94
John sought the advice of a friend after his wife left him.	1.64	7.24	3.51	4.67
John spoke to a friend about his marriage troubles.	0.70	7.15	3.46	4.81
John met with a lawyer after his wife suggested they get a divorce.	0.94	7.37	3.81	5.53
John sent text messages to his best friend’s wife.	-0.82	6.49	4.63	6.08
John went to the dry cleaner to have lipstick removed from his collar.	-0.24	5.86	4.71	5.75
John was spotted with his best friend’s wife in a hotel lobby.	-1.85	6.14	5.45	6.17
John went out for a coffee with his best friend’s wife.	-0.60	6.14	4.55	5.90
John found out that his best friend’s wife was pregnant with his baby.	-3.08	5.74	6.74	6.28
**Scenarios related to dog-target (Experiments 2)**				
John accompanied his wife to the vet to have his dog treated for a head injury.*	2.65	7.20	3.21	4.40
John took his dog to obedience training as it would not come when called.	1.79	7.15	2.99	4.39
John got up multiple times during the night to check on his dog, which had a head injury.	3.02	6.91	2.85	3.72
John took his dog for a walk at the local dog park.	2.14	7.60	2.35	4.01
John injured his foot while walking his dog.	0.26	6.76	3.36	4.58
John arranged for someone to look after his dog while he was at work, after noticing his dog had a bump on its head.	2.43	6.40	2.82	3.63
John bought an extendable lead for his dog as it would not come when called.	0.67	6.39	3.16	4.42
John sometimes kicks things when he loses his temper.	-1.67	6.81	4.72	6.19
**Scenario related to tree-target (Experiment 3)**				
John enjoys uninterrupted views of the river from his living room now that his neighbors’ tree has been removed.*	0.28	6.78	3.95	5.60

Note. For the Morality and Believability ratings ‘John’ was replaced with Person 1, 2 etc.
* denotes the selected coherence-building statement.
