# Supplementary material for: Did he or didn’t he? Mixed evidence for the continued influence of retracted misinformation on person impressions
Source: PLoS One. 2025 May 7;20(5):e0322045. doi: 10.1371/journal.pone.0322045 (PMC12058158; doi:10.1371/journal.pone.0322045)
Supplement: S2 File — (PDF) [file pone.0322045.s002.pdf]

S2 File. Impression Formation Task

S1 Table. Neutral Behavior Statements Presented in Initial Behavior Set (Phase 1)

Neutral Statement	Morality (−4 to 4)	Believability (0 to 8)
John brought in the groceries from the car and dropped one of the bags, which caused the eggs to break.	0.07	7.22
John ordered his favorite dish from a Chinese restaurant.	0.22	7.66
John went to purchase a new pair of shoes but couldn't find any that were comfortable.	0.21	7.07
John went to a friend's house to play a card game.	0.44	7.46
John ran out of paint when painting his home and had to go the hardware store.	0.15	7.31
John is learning French as he always wanted to learn another language.	0.57	7.22
John prepared a roast chicken and made the stuffing from scratch.	0.35	7.31
John learned how to play the piano when he was a child.	0.51	7.38
John tried to patch a puncture in the wheel of his bike but couldn't, so he purchased a new tube instead.	0.34	7.18
John locked his keys in the house and had to call a locksmith.	0.19	7.23
John was unable to fix the dripping faucet so he had to call the plumber.	0.38	7.29
John arrived at the airport only to discover he had left his passport at home.	0.06	7.03

S2 Table. Neutral Behavior Statements Presented in Updated Behavior Set (Phase 2)

Neutral Statement	Morality (−4 to 4)	Believability (0 to 8)	Type
John was forced to pay multiple bank fees for not paying his credit card repayment on time.	−0.40	7.08	Addition
John sneezed loudly in an important meeting.	−0.11	7.10	Addition
John did really well at the quiz night.	0.41	7.27	Addition
John solved a crossword puzzle in the newspaper.	0.11	7.26	Addition
John ordered a take-away coffee but spilled it when he tried to take a sip.	0.08	7.33	Addition
John did not meet his sales targets for the month at work.	0.06	7.08	Addition
John was singing loudly to his favorite song in the car.	0.49	7.38	Retraction
John ate his lunch and went back to work with food stuck in his teeth.	−0.02	7.28	Retraction (No-Retraction conditions only)
John was running late so he drove to work rather than taking the bus.	0.40	7.07	Confirmation
John played chess with his friend, winning the game.	0.30	7.37	Confirmation

Note. Addition indicates behavior statement was added as part of Phase 2 (not presented in Phase 1). Retraction indicates behavior statement from the initial behavior set was retracted. Confirmation indicates a behavior statement from the initial behavior set was confirmed.

S3 Table. Target Statements Across Experiments

Target Statement	Morality (−4 to 4)	Believability (0 to 8)	Valence	Experiment
John had an affair with his best friend’s wife.	−3.40	6.84	Negative	1
John kicked his pet dog hard in the head when it didn’t come when called.	−3.55	6.79	Negative	2
John cut down his neighbors' chestnut tree while they were out, as he was fed up with it obstructing his view.	−3.27	5.11	Negative	3
John went to a fancy restaurant but couldn’t pronounce the items on the menu.	0.06	6.75	Neutral	1-3

S1 Text. Likability Score

The Reysen Likability Scale (Reysen, 2005) was used to measure the likability of "John". Participants responded to each item on a seven-point Likert scale ranging from 1 (*very strongly disagree*) to 7 (*very strongly agree*). Four reverse-coded items were added to allow for identification of inconsistent responders and were not used in the likability analysis (though including them did not alter the results). Reverse-coded items are marked with a *.

1. This person is friendly.
2. This person is likeable.
3. This person is warm.
4. This person is unpleasant.*
5. This person is approachable.
6. I would ask this person for advice.
7. I would like this person as a co-worker.
8. I would like this person to stay away from me.*
9. I would like this person as a roommate.
10. I would like to be friends with this person.
11. This person is annoying.*
12. This person is physically attractive.
13. This person is cold.*
14. This person is similar to me.
15. This person is knowledgeable.

S2 Text. Recognition Test 1: Initial Behavior Questions

Participants were required to select the correct answer out of three choices. Question 2 was based on the target statement, so it varied depending on the experimental condition and Experiment, with the remaining questions (1, 3–8) the same across experimental conditions. Correct answer are marked with a *.

1. John had to call the plumber because he couldn't fix…?
a. The dripping faucet*
b. The low water pressure
c. The clogged toilet

2. What couldn't John do at the restaurant? *(Neutral, All Experiments)*
a. Find the restroom
b. Pronounce the menu items*
c. Look his date in the eye

2. Who did John have an affair with? *(Negative No-Retraction, Negative Retraction, Experiment 1)*
a. His best friend's sister
b. His best friend's wife*
c. His best friend's mother
2. Which pet did John kick? *(Negative No-Retraction, Negative Retraction, Experiment 2)*
a. His pet cat

b. His pet dog*
c. His pet bird

2. What did John do while his neighbors were out? *(Negative No-Retraction, Negative Retraction, Experiment 3)*
a. Smash all their windows
b. Cut down their chestnut tree*
c. Set fire to their car

3. Why did John drive to work rather than taking the bus? *(Experiments 2 and 3)*
a. He was running late.*
b. The bus smells bad.
c. His nemesis was on the bus.

4. When John dropped a bag of groceries, what broke?
a. A jar of spaghetti sauce
b. Eggs*
c. His spirit

5. What language is John learning?
a. Russian
b. Japanese
c. French*

6. When John got to the airport, what had he left at home?
a. His wallet
b. His passport*
c. His luggage

7. What game did John play against his friend, and win?
a. Poker
b. Tennis
c. Chess*

8. What did John try to purchase, but couldn't find any that were comfortable?
a. Pants
b. Shoes*
c. Sunglasses

S3 Text. Recognition Test 2: Updated Information Questions

Participants were required to select the correct answer out of four choices. Question 2 varied across experiments and depending on the experimental condition (because different statements were retracted in different conditions, and an alternative response was based on a statement that was not used in all experiments), with the remaining questions (1 and 3) the same across experiments and experimental conditions. * indicates correct response.

1. Which of these behaviors were confirmed?

a. John was running late so he drove to work rather than taking the bus.*

b. John brought in the groceries from the car and dropped one of the bags, which caused the eggs to break.

c. John played chess with his friend, winning the game.

d. John locked his keys in the house and had to call a locksmith.

2. Which of these behaviors were retracted?

a. John ate his lunch and went back to work with food stuck in his teeth.* *(Negative No-Retraction)*

a. John had an affair with his best friend's wife. *(Negative Retraction, Experiment 1)*

a. John kicked the dog hard in the head when it didn't come when called. (*Negative Retraction, Experiment 2*)

a. John enjoys uninterrupted views of the river from his living room now that his neighbors' tree has been removed. (*Negative Retraction, Experiment 3*)

a. John went to a fancy restaurant but couldn't pronounce the items on the menu. *(Neutral)*

b. John prepared a roast chicken and made the stuffing from scratch.

c. John went to a friend's house to play a card game.

d. John was singing loudly to his favourite song in the car.

Which of these behaviors were added?

a. John arrived at the art exhibition early so he could view the collection before it got too busy.

b. John did really well at the quiz night.*

c. John was forced to pay multiple bank fees for not paying his credit card repayment on time.

d. John told the children to be quiet in the library.

S4 Text. Inference Questions Experiment 1.
As part of the study, you were presented with behaviors that John had engaged in. Based on this information please answer the following questions.

1. If you could tell someone about one specific thing John has done what would it be? [open-ended response] [NOTE: infQ1 in dataframe]

Please rate how much you endorse the following statements from 0 (strongly disagree) to 10 (strongly agree).

2. John is an honorable man. [0 (*Strongly Disagree)* – 10 (*Strongly Agree)*]* [infQ3]
3. John is a good friend. [0 (*Strongly Disagree)* – 10 (*Strongly Agree)*]* [infQ4]
4. I would be surprised if John had a fight with his best friend. [0 (*Strongly Disagree)* – 10 (*Strongly Agree)*]* [infQ5]
5. John should be ashamed of his behavior. [0 (*Strongly Disagree)* – 10 (*Strongly Agree)*] [infQ6]
6. John's best friend should reconsider his friendship with John. [0 (*Strongly Disagree)* – 10 (*Strongly Agree)*] [infQ7]
7. Describe briefly in one sentence what kind of relationship John has with his best friend's wife. [open-ended response] [infQ2]

Note. * denotes reverse-scored item.

S5 Text. Explicit Target-Behavior Question

1. How likely is it that John had an affair with his best friend's wife? [0 (E*xtremely Unlikely*), to 10 (*Extremely Likely*). [open-ended response] [infQ8]

S6 Text. Inference Open-ended Scoring Criteria Experiment 1

Misinformation Reference (MR) = 1. A MR of 1 will be given for any unambiguous references to the misinformation or any references that clearly imply misinformation belief. Some examples include:

- Many thought/many believe
  - "Many believe John was having an affair"
- Likely / most likely / very likely / it is believed
  - "It's likely John and his best friend's wife were having an affair"
  - "It's believed John was having an affair"
- Specific mentions of the misinformation or any words unequivocally relating to John having an affair with his best friend's wife.
  - "having an affair"

  - "extra-marital affair"
  - "sexual relationship"
  - "intimate relationship"
  - "romantic relationship"
  - "secret relationship"
  - "sleeping with his best friend's wife"
  - "cheating with his best friend's wife"
  - "inappropriate relationship"
- Misinformation is clearly used to infer judgement about John's character.
  - "he is seeing his best friend's wife, that's disgraceful"
  - "adulterer"
  - "untrustworthy"
  - "dishonest"
  - "cheater"
  - "betrayed"
- Misinformation is clearly used to infer judgement about John and his best friend's wife relationship.
  - "clearly not a good relationship given the affair"

MR = 0.5. A MR of 0.5 will be allocated to ambiguous references to the misinformation.

- Mentions of the misinformation or any words relating to John having an affair with his best friend's wife are ambiguous.
  - "close relationship" or "very close" or "too close"
  - "suspicious relationship"
  - "shady, secretive"
  - "sketchy"
  - "wrong"
- Other responses which express any level of doubt from the participant.
  - Could
  - Possible
  - May / might
  - Allegedly
  - Potential
  - Presumably
  - Probably
  - Appears
  - Somewhat
  - Seems to
  - Seemingly
  - Suspected
  - Speculated
  - Guessing
- Response questions belief in misinformation.
  - An affair maybe?
- It is ambiguous as to whether misinformation is used to infer John's character.
  - "sleezy"

    - Flirt / flirtatious
- It is ambiguous as to whether misinformation is used to infer judgement about John and his best friend's wife relationship.
    - "too friendly"

MR equal to 0

A MR score of 0 will be awarded when there is no misinformation referenced in the response, including implied belief in the misinformation. Scores will be awarded as per the criteria set below.

- The response recalls the behavior statements accurately and shows no suggestion that the participant believes the misinformation or relied upon the misinformation in their reasoning.
    - "It was mentioned that John was having an affair, but that was retracted."
    - "they never had an affair"
- If response references 'a friendship or relationship' without any reference to the misinformation.
    - "They were just friends."
    - "close friendship"
    - "They knew each other, but they were not together."
    - "Good relationship"
    - "Casual relationship"
    - "Cordial relationship"
- If misinformation has not been used at all to infer judgement about John's character.
    - "friendly"
    - "nice guy"
    - "lazy"
    - "unpredictable"

S7 Text. Inference Questions Experiment 2

As part of the study, you were presented with behaviors that John had engaged in. Based on this information please answer the following questions.

1. If you could tell someone about one specific thing John has done what would it be? [open-ended response] [NOTE: infQ1]

Please rate how much you endorse the following statements from 0 (strongly disagree) to 10 (strongly agree).

2. John is a kind-hearted man. [0 (*Strongly Disagree)* – 10 (*Strongly Agree)*]* [infQ3]
3. John is a good person. [0 (*Strongly Disagree)* – 10 (*Strongly Agree)*]* [infQ4]
4. I would be surprised if John ever lost his temper. [0 (*Strongly Disagree)* – 10 (*Strongly Agree)*]* [infQ5]
5. John should be ashamed of his behavior. [0 (*Strongly Disagree)* – 10 (*Strongly Agree)*] [infQ6]
6. John's wife should be concerned about John's behavior. [0 (*Strongly Disagree)* – 10 (*Strongly Agree)*] [infQ7]
7. Describe briefly in one sentence how you think John's pet dog could have received a head injury? [open-ended response] [infQ2]

Note. * denotes reverse-scored items.

S8 Text. Inference Open-ended Scoring Criteria Experiment 2

Misinformation Reference (MR) = 1.

A MR of 1 will be given for any unambiguous references to the misinformation or any references that clearly imply misinformation belief. Some examples include:

- Many thought/many believe
  - "Many believe John kicked the dog in the head"
- Could have
  - "could have been that John kicked the dog in the head"
- Likely / most likely / very likely / it is believed
  - "It's likely John kicked his dog in the head"
  - "It's believed John kicked the dog in the head"
- Specific mentions of the misinformation or any words unequivocally relating to John kicking the dog when it didn't come when called.
  - "John kicked the dog"
  - "John hurt the dog"
  - "he injured the dog"
  - "he attacked/was violent towards his dog"
- Misinformation is clearly used to infer judgement about John's character.
  - "he hurt his dog that is disgraceful"
  - "evil man"
  - "what he did to the dog was wrong"
  - "cruel man"
  - "abuses animals"
  - "violent"
  - "aggressive"
- Misinformation is clearly used to infer judgement about what caused the dog's head injury.
  - "clearly he caused the head injury given he kicked the dog."

MR = 0.5

A MR of 0.5 will be allocated to ambiguous references to the misinformation.

- Mentions of the misinformation or any words relating to John kicking the dog hard in the head are ambiguous.
  - "mean to his dog"
  - "not nice towards his dog"
  - "got upset with his dog"
  - "got cross with his dog"
- Other responses which express any level of doubt from the participant.
  - May / might
  - Allegedly
  - Potential
  - Presumably
  - Probably
  - Appears
  - Somewhat
  - Seems to
  - Seemingly
  - Suspected
  - Speculated
  - Guessing

- Response questions belief in misinformation.
  - He kicked the dog, maybe?
- It is ambiguous as to whether the misinformation is used to infer John's character.
  - "loses his temper".
  - "gets upset"
- It is ambiguous as to whether misinformation is used to infer judgement about the cause of the dog's head injury.
  - "he may have hurt his dog"
  - "it probably was John who caused the head injury"

MR equal to 0

A MR score of 0 will be awarded when there is no misinformation referenced in the response, including implied belief in the misinformation. Scores will be awarded as per the criteria set below.

- The response recalls the behavior statements accurately and shows no suggestion that the participant believes the misinformation or relied upon the misinformation in their reasoning.
  - "It was mentioned that John had kicked the dog in the head, but that was retracted."
  - "he never kicked the dog"
  - "he did not hurt the dog"
- If response details the cause of the dog's head injury without any reference to the misinformation.
  - "the dog got hit by a car."
  - "it is unknown how the dog received the head injury"
  - "perhaps the dog got attacked by another dog"
  - "it was an accident"
  - "it was John's carelessness"
- If misinformation has <u>not</u> been used at all to infer judgement about John's character.
  - "friendly"
  - "loyal"
  - "nice guy"
  - "lazy"
  - "unpredictable"

S1 Figure. Final Impression Rating Measure (Experiments 2 and 3)

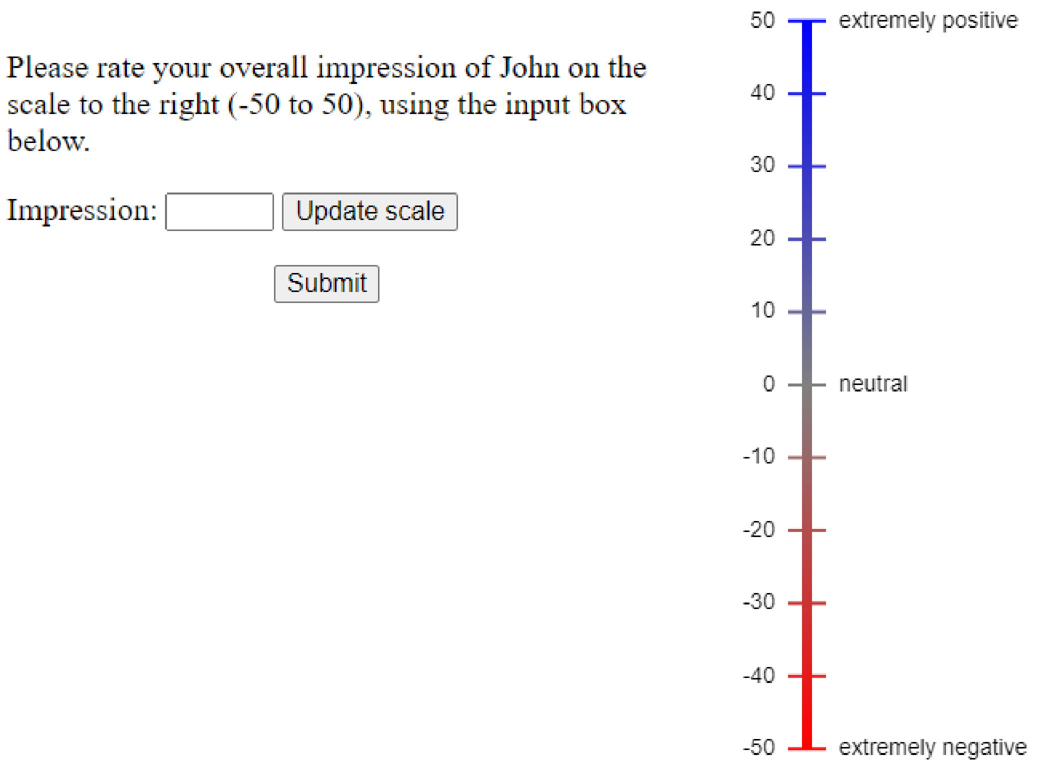

S9 Text. Inference Questions Experiment 3

As part of the study, you were presented with behaviors that John had engaged in. Based on this information please answer the following questions.

1. If you could tell someone about one specific thing John has done what would it be? [open-ended response] [NOTE: infQ1 in dataframe]

Please rate how much you endorse the following statements from 0 (strongly disagree) to 10 (strongly agree).

2. John is a good person. [0 (*Strongly Disagree)* – 10 (*Strongly Agree)*]* [infQ3]
3. John has never trespassed on another person's property. [0 (*Strongly Disagree)* – 10 (*Strongly Agree)*]* [infQ4]
4. John's neighbors have reason to be upset with John. [0 (*Strongly Disagree)* – 10 (*Strongly Agree)*] [infQ5]
5. John has no respect for other people. [0 (*Strongly Disagree)* – 10 (*Strongly Agree)*] [infQ6]

6. The police should charge John. [0 (*Strongly Disagree)* – 10 (*Strongly Agree)*]

   [infQ7]

7. Describe briefly in one sentence what happened to John's neighbors' chestnut tree?

   [open-ended response] [infQ2]

*****denotes item is reverse-scored

S10 Text. Inference Open-ended Scoring Criteria Experiment 3

Misinformation Reference (MR) = 1.

A MR of 1 will be given for any unambiguous references to the misinformation or any references that clearly imply misinformation belief. Some examples include:

- Many thought/many believe
  - "Many believe John cut down the tree"
- Likely / most likely / very likely / it is believed
  - "It's likely John chopped down the neighbors' tree"
  - "It's believed John cut down his neighbors' beloved chestnut tree"
- Specific mentions of the misinformation or any words unequivocally relating to John cutting down his neighbors' beloved chestnut tree.
  - "he chopped it down"
  - "John cut down his neighbors' tree"
  - "it was John who removed the tree"
- Misinformation is clearly used to infer judgement about John's character.
  - "he chopped down his neighbors' beloved chestnut tree, that's disgraceful"
  - "evil"
  - "can't be trusted"
- Misinformation is clearly used to infer judgement about John and him cutting down his neighbors' tree.
  - "clearly not a good person given he chopped down his neighbors' tree"

MR = 0.5

A MR of 0.5 will be allocated to ambiguous references to the misinformation.

- Mentions of the misinformation or any words relating to John cutting down his neighbors' tree are ambiguous.
  - "tree was damaged"
  - "suspicious of John"
  - "it was wrong what John did"
- Other responses which express any level of doubt from the participant.
  - Could
  - Possible
  - May / might

    - Allegedly
    - Potential
    - Presumably
    - Probably
    - Appears
    - Somewhat
    - Seems to
    - Seemingly
    - Suspected
    - Speculated
    - Guessing
- Response questions belief in misinformation.
    - John cut it down maybe?
- It is ambiguous as to whether misinformation is used to infer John's character.
    - "mean spirited"
    - "not a very nice person"
- It is ambiguous as to whether misinformation is used to infer judgement about John cutting down his neighbors' tree.
    - "he can't be trusted"

MR equal to 0

A MR score of 0 will be awarded when there is no misinformation referenced in the response, including implied belief in the misinformation. Scores will be awarded as per the criteria set below.

- The response recalls the behavior statements accurately and shows no suggestion that the participant believes the misinformation or relied upon the misinformation in their reasoning.
    - "It was mentioned that John cut down his neighbors' tree, but that was retracted."
    - "he did not cut down the tree"
    - "he cut it down, but then they said it didn't happen"
- If response references 'the tree being removed' without any reference to the misinformation.
    - "It got cut down."
    - "the neighbor chopped it down"
    - "the tree was removed."
- If misinformation has not been used at all to infer judgement about John's character.
    - "friendly"
    - "trustworthy"
    - "good person"

References

Reysen, S. (2005). Construction of a new scale: The Reysen Likability Scale. *Social Behavior and Personality: An International Journal*, *33*(2), 201–208. https://doi.org/10.2224/sbp.2005.33.2.201
