# Supplementary material for: Did he or didn’t he? Mixed evidence for the continued influence of retracted misinformation on person impressions
Source: PLoS One. 2025 May 7;20(5):e0322045. doi: 10.1371/journal.pone.0322045 (PMC12058158; doi:10.1371/journal.pone.0322045)
Supplement: S3 File — (PDF) [file pone.0322045.s003.pdf]

S3 File. Additional Analyses

S1 Text. Experiment 1 Additional Analyses

Explicit Target-Behaviour Score

Explicit target-behaviour scores supported observations made based on the likability scores and inference scores. Explicit target-behaviour scores were entered into a linear regression with condition as a predictor (negative retraction; negative no-retraction; neutral). The model was significant, $F(2, 298) = 166.20$, $p < .001$, $R^2 = .53$. Explicit target-behaviour scores were higher (i.e., participants made more references to the negative misinformation) for the negative no-retraction condition compared to the neutral condition, $\beta = -5.20$, $SE = 0.33$, $t = -15.96$, $p < .001$, and compared to the negative retraction condition, $\beta = -5.68$, $SE = 0.35$, $t = -16.12$, $p < .001$. There was no evidence of a statistical difference between the negative retraction condition and neutral condition, $\beta = -0.48$, $SE = 0.36$, $t = -1.34$, $p = .183$. This suggests that the retraction was fully effective and there was no evidence of a CIE when there was explicit reference made to the misinformation.

S2 Text. Additional Analyses Experiment 2

Final Impression Ratings

Final impression ratings were entered into a linear regression with condition as a predictor (negative retraction; negative no-retraction; neutral). The model was significant, $F(2, 297) = 143.70$, $p < .001$, $R^2 = .49$. Final impression ratings were lower for the negative no-retraction condition compared to the neutral condition, $\beta = -45.99$, $SE = 3.02$, $t = -15.21$, $p < .001$, and compared to the negative retraction condition, $\beta = 39.16$, $SE = 3.20$, $t = 12.22$, $p < .001$. There was evidence of a statistical difference between the negative retraction condition and neutral condition, $\beta = -6.84$, $SE = 2.54$, $t = -2.70$, $p = .008$. This suggests the retraction was not fully effective and there was evidence of a CIE in final impression ratings.

S3 Text. Experiment 3 Additional Analyses

Final Impression Rating

Final impression ratings were entered into a linear regression with condition as a predictor (negative retraction; negative no-retraction; neutral). The model was significant, $F(2, 298) = 32.09$, $p < .001$, $R^2 = .18$. Final impression ratings were lower for the negative no-retraction condition compared to the neutral condition, β = −19.74, *SE* = 3.29, *t* = −5.91, $p < .001$, and compared to the negative retraction condition, β = –23.18, *SE* = 3.29, *t* = –7.04, $p < .001$. There was no evidence of a statistical difference between the negative retraction condition and neutral condition, β = 3.76, *SE* = 2.69, *t* = 1.40, *p* = .164. This suggests the retraction was fully effective and there was no evidence of a CIE in final impression ratings.

S1 Table. Summary of Equivalence Tests for Likability Scores and Inference Scores for Experiments 1 and 3

	Small Effect (±0.1)	
	$t_{equ}(df)$	p_{equ}
Experiment 1		
Negative Retraction vs. Neutral (Likability)	–1.36 (299)	.663
Negative Retraction vs. Neutral (Inference)	1.77 (299)	.835
Experiment 3		
Negative Retraction vs. Neutral (Likability)	0.73 (299)	.377
Negative Retraction vs. Neutral (Inference)	0.17 (299)	.094

Note. t_{equ} / p_{equ} = Equivalence Test. * indicates p < .05 and is evidence for the null hypothesis.
