# Supplementary material for: Did he or didn’t he? Mixed evidence for the continued influence of retracted misinformation on person impressions
Source: PLoS One. 2025 May 7;20(5):e0322045. doi: 10.1371/journal.pone.0322045 (PMC12058158; doi:10.1371/journal.pone.0322045)
Supplement: Attachment — Submitted filename: Response to PLOS ONE Reviewers.doc [file pone.0322045.s005.doc]

School of Psychological Science

35 Stirling Highway, CRAWLEY

Perth, Western Australia, WA 6009

T	+61 (0)8 6488 2688

F	+61 (0)8 6488 1006

E	nicolas.fay@gmail.com

CRICOS Provider Code: 00126G

28 February 2025

Prof Alessandra S. Souza

Academic Editor – PLOS ONE

Dear Prof Souza,

Thank you for reviewing our manuscript, "Did he or didn’t he? Mixed evidence for the continued influence of retracted misinformation on person impressions". We are encouraged by the positive assessment, and welcome the reviewer feedback. The manuscript has been revised considering these comments. The feedback and our responses are detailed below, first in response to the Editor and then to Reviewer 1:

**Editor**

1. I did not understand the logic of the addition and the confirmation phrases. Why are they considered an addition or confirmation? I couldn't see any other phrase they were related to in the supplement. Please be more clear about the logic behind the methodology used.

We thank the editor for pointing this out. To improve understanding, we have adjusted the wording to make clear the use of the addition and confirmation statements and provided examples of each, “The second phase presented 10 updates; these included six additional neutral filler statements not presented in the first phase (e.g., “ADDITION: John did really well at the quiz night”), two confirmations of neutral filler statements from the first phase (e.g., “CONFIRMATION: John was running late, so he drove to work rather than taking the bus”), and two retractions of statements from the first phase. In the negative retraction and neutral conditions, this included the retraction of the respective target statement (e.g., “RETRACTION: John did NOT have an affair with his best friend’s wife”), as well as a retraction of a neutral filler statement (e.g., “RETRACTION: John was NOT singing loudly to his favorite song in the car”). In the negative no-retraction condition, two neutral filler statements were retracted.” (see p9, lines 213-220). In addition we have added to the supplementary materials in S2 File p.2 stating “Note. Addition indicates behavior statement was added as part of Phase 2.”

2. Regarding the data-analysis, you try to establish that there is no difference between the retraction and control conditions based on a non-significant p-value. This is not appropriate. Please include targeted analyses that can be used to establish that two conditions do not differ (e.g., Bayesian analysis; equivalence tests).

An equivalence analysis has now been included and can be found in the results section, p.16 (starting line 354) and p. 25 (starting line 532).

3. I was not convinced about the meta-analysis. I was under the impression at first that the meta-analysis included the articles previously published by the group (which seems to comprise most of the literature on this topic), but upon re-reading, I realized that it only included the 3 reported studies. Why not include all the available evidence on CIE with person impressions?

We have rerun the meta-analysis using the available evidence on the CIE in person impressions based on the present study and the four experiments from Mickelberg, Walker, Ecker, Howe et al. 2024. This has now been updated. See p.26 lines 565-569.

4. As mentioned in the discussion, in Experiment 2, the average rating was close to 3 for the negative no-retraction group; in the other experiments, ratings were close to 4, which suggest people were more neutral. I wonder whether this indicates that the effect is moderated by the degree with which the misinformation was able to affect the formation of an impression. I believe more data is needed to address which conditions led to persistent effects on impressions. Perhaps including targets that are higher in emotionality (e.g., messing with the pension funds of the company; domestic violence; sexual harassment) could led to observations of persistent effects. This was not considered here as a potential moderator (this might help explain why in some prior studies a CIE was observed?), but it would be important in order to establish that retractions are more effective for person impressions.

We appreciate the editor's suggestion for additional testing. However, we would like to clarify that Experiment 3 aimed to address this aspect of our research question and determine the generalisability of our results. Rather than conducting further testing at this stage, we have strengthened the discussion to more fully explore potential moderating factors, including emotionality. We believe this approach provides a comprehensive framework for future research, outlining potential moderating factors that require systematic testing under controlled conditions. This expanded discussion sets a clear direction for subsequent studies in this field (see p27-p31). We also note that while highly emotional or extreme content might lead to a stronger CIE in person impressions, this may not represent typical social interactions, potentially reducing the broader applicability of results.

**Reviewer 1**

1. Experimentation, Statistical Methods, and Technical Standards
   P. 23: The paper mentions that the CIE was only detected in Experiment 2 and that the effect sizes were small. This discrepancy across experiments could be due to various factors, such as differences in the nature of the misinformation or the emotional content. I recommend expanding this section to address how different types of misinformation (e.g., moral vs. non-moral misinformation) might lead to stronger or weaker effects on person impressions.

We have emphasised the need to clarify which characteristics would lead to a stronger or weaker CIE on person impressions based on Reviewer 1’s suggestion, including an investigation of moral vs. non-moral misinformation, moral sub-dimensions and content that varies in emotionality.

“Additionally, misinformation statements that are more extreme often elicit stronger negative reactions [1,63–65], potentially influencing the strength of the CIE in person impressions. While a focus on more extreme cases would limit generalizability, it is important for future work to make controlled comparisons of various moral behaviours and the dimensions they tap into [60,66] to conclusively determine their impact on the CIE in person impressions.” (p29, lines 625-629).

Please also see our response to the Editor at point 4 and our response to Reviewer 1 at point 5.

2. P. 23: The authors suggest that dynamic vs. non-dynamic task designs may explain the differing results across experiments. However, to make a definitive claim, the authors should consider replicating the study with the same vignettes but varying the task design. This would offer more direct evidence for the impact of task design on the CIE. Without this, the suggestion remains speculative.

We appreciate the Reviewer’s suggestion to replicate the study with the same vignettes while varying the task design. We had the opportunity to address this in our previous work (Mickelberg, Walker, Ecker, Howe et al., 2024) where the target materials from Experiments 3 and 4 were identical to Experiments 1 and 2 used in the current paper. The mixed findings suggest the nature of the task itself cannot explain the observed results (see p. 28, lines 604-605).

3. Testable Hypotheses and Scientific Contribution
   P. 2: The hypotheses are clearly articulated, and the study addresses an important gap in the literature. However, the mixed results across the experiments raise questions about the robustness of the CIE. I recommend the authors provide a deeper analysis of these inconsistencies. The manuscript could benefit from more discussion on the potential reasons for these mixed results, including moral dimensions of the misinformation and counterfactual thinking.

P. 23: I also suggest the authors reference O'Rear & Radvansky (2020), which argues that the failure to accept retractions is related to the belief in the correction. This could provide additional theoretical context for explaining why the misinformation persisted in some experiments but not others. Additionally, exploring counterfactual thinking (how difficult it was for participants to generate alternatives to the misinformation) could help explain the variability in CIE across the experiments.

We have expanded and strengthened our discussion to address the mixed results and the robustness of the CIE in person impressions (see discussion p27-31). This includes greater emphasis on the potential explanations for the variability in the CIE in person impressions across our experiments. Of note, we have expanded discussion on the believability of retractions and the ability to generate alternative explanations, such as counterfactual thinking, and reference O’Rear & Radvansky (2020).

“While the believability of the misinformation statement was taken into consideration, future work may wish to manipulate the credibility of the retraction, as this may influence the observation of the CIE in person impressions. Research has shown that the continued influence effect can occur when people fail to accept the retraction [63], although it should be noted that even with highly credible corrections, the CIE has been shown to reliably occur [see 18]. Individual differences may also influence the effectiveness of retractions. For instance, individuals with a counterfactual mindset—that is, those with the ability to generate alternative explanations—are more likely to revise their beliefs when presented with contradictory evidence [64]. Future research would benefit from exploring the myriad drivers that may influence a retraction’s effectiveness as it relates to person impressions.” (p. 29, lines 635-644).

In addition, please see our response to the Editor point 4 and our response to Reviewer 1 at point 5.

4. Appropriateness of the Design and Methods
   P. 7: The coherence-building elements used in the experiments are mentioned, but the manuscript does not provide enough detail on how these elements were chosen. The authors should elaborate on the selection process. Specifically, the criteria that coherence-building statements were "unlikely to imply" but "likely to explain" the misinformation are not clearly explained (lines 239–240). This criterion should be better justified.

The criterion and how these elements have been chosen are further elaborated upon. This can be viewed on p11 lines (253-258). “These criteria were chosen because the coherence-building statement was required to be neutral so as not to have much direct influence on impressions, and highly believable so as not to be ignored. In addition, the coherence-building statement should not independently imply the target statement in its absence (low ‘imply’ rating) as this would interfere with the neutrality of the control condition, but should provide a logical explanation for when the target statement was presented (high ‘explain’ rating).”

5. P. 23 (lines 542–544): The authors note that the misinformation in Experiment 2 involved an extreme act of violence (kicking a pet dog), which may have tapped into a different moral dimension compared to other misinformation (e.g., dishonesty). I suggest the authors explore the moral dimensions of misinformation more thoroughly and compare it to other studies. This could include comparisons to prior work like Ecker & Rodricks (2020) where vignettes seem to include violent behaviour (e.g. John slapped his girlfriend during an argument) (lines 493–504). If the moral dimension should be driving the CIE should this vignette not be more likely to show evidence of continued reliance on the retracted claim?

We thank Reviewer 1 for raising this point. We have included comparisons to prior work as per Reviewer 1’s suggestion and how behaviours which activate different moral dimensions may contribute to the results observed.

“Future work could explore whether the CIE in person impressions is dependent on misinformation statements that tap into specific moral dimensions [43,59,60] compared to non-moral dimensions [61,62]. For example, certain moral and violent behaviours (“…kicked a dog hard in the head…”; Experiment 2) may activate moral foundations [e.g., care vs. harm; 59] that potentially lead this behaviour to be perceived as particularly diagnostic of a person’s character [8], making a CIE more likely to arise. In contrast, other morally questionable and violent behaviours [e.g., “slapped his girlfriend during an argument”; 12] may tap into different moral dimensions [e.g., loyalty vs. disloyalty; 59], potentially explaining the mixed results, although this interpretation is speculative given the two behaviours appear similar at face value. Additionally, misinformation statements that are more extreme often elicit stronger negative reactions [1,63–65], potentially influencing the strength of the CIE in person impressions. While a focus on more extreme cases would limit generalizability, it is important for future work to make controlled comparisons of various moral behaviours and the dimensions they tap into [60,66] to conclusively determine their impact on the CIE in person impressions.” (p28-29, 616-629).

Please also see response to Reviewer 1 point 3, and the editor’s point 4.

6. P. 19: The authors conduct an internal meta-analysis. However, it is unclear why the random-effects model was chosen over other models, and how this decision affects the generalizability of the findings. I recommend more discussion of the rationale behind choosing this model and its implications for interpreting the results.

We have added that due to variability in effect sizes a random-effects model was chosen (see p.26, line 557-558).

7. Data Presentation and Conclusion
   P. 26: The authors conclude that there is no conclusive evidence for the CIE in person impressions, which is somewhat understated in light of the mixed results. Given the mixed findings, the conclusion should engage more deeply with the broader literature on belief perseverance and misinformation correction. This would allow the authors to emphasise the implications of their findings, such as how misinformation can impact person impressions in everyday life or online interactions.

This point is well taken. We have reworked and strengthened the discussion (p.27-31) highlighting the importance of future work that explores the specific conditions necessary for the CIE in person impressions whilst also drawing from the broader literature on belief perseverance and misinformation correction.

Furthermore, we have strengthened the conclusion to reflect the broader implications of our mixed results. “To clarify the mixed results, future research should examine the factors that may moderate the emergence of a continued influence in person impressions, such as the specific attributes of the misinformation or the presence of social category information. This may reveal the mechanisms underlying the continued influence effect in person impressions, informing strategies to combat person-related misinformation.” (see p. 32, lines 701-706).

Please also see response to Reviewer 1 point 1 and 3.

8. Writing and Clarity
   The manuscript is generally well-written. However, the results section could benefit from clearer transitions between experiments and findings, especially in Experiment 2. The mixed results are not immediately clear, and it would help to clarify why Experiment 2 produced a different outcome compared to the other experiments.

Based on this feedback, we have emphasised the mixed results by comparing how Experiment 2’s findings contrast with the results in Experiment 1 (see p. 21, lines 455-456) and Experiment 3 (see p. 26, lines 548-551) which states “The results of Experiments 1 and 3 contrast with the positive results reported in Experiment 2 which found evidence of a CIE in person impressions. Overall, the findings from the three experimental studies found mixed evidence for the CIE in person impressions.”

9. Title, Abstract, and Figures
   Abstract: The abstract could benefit from a more explicit mention of the mixed results observed across the three experiments. It would be helpful to briefly outline the implications of these mixed results for future research or real-world applications.

This suggestion is well taken. We have emphasised the mixed results and briefly outlined the implications of the mixed results in the abstract, stating “The mixed findings indicate that, unlike retracted event-related misinformation, retracted person-related misinformation does not consistently show a continued influence effect. Future research should investigate potential moderating factors, such as the attributes of the misinformation and the presence of social-category information about the protagonist, to reveal the mechanisms underlying the continued influence effect in person impressions.” (see p2, lines 34-39).

10. Title, Abstract, and Figures

Figures: The figures are well-organised and support the data effectively. However, in Experiment 1, the error bars and bootstrapped confidence intervals are not sufficiently explained. I suggest including a more detailed description of these statistical details in the figure captions. For example, the number of resamples used in the bootstrapping process should be mentioned to enhance clarity.

Thank you for this helpful suggestion. We have added the number of resamples used in the bootstrapping process stating “error bars are 95% bootstrapped confidence intervals (1000 resamples)” (see p16, line 352).

11. References
    The references are well-selected, but the authors should ensure consistency in citation formatting, particularly with the journal names and issue numbers. For example, entries such as "Ecker UKH, Lewandowsky S, Cook J, et al." should be reviewed for consistency with the PLOS ONE citation style guide.

The references are now in line with PLOS ONE citation guidelines.

**Journal Requirements**

1. Please ensure that your manuscript meets PLOS ONE's style requirements, including those for file naming. The PLOS ONE style templates can be found at <https://journals.plos.org/plosone/s/file?id=wjVg/PLOSOne_formatting_sample_main_body.pdf> and [https://journals.plos.org/plosone/s/file?id=ba62/PLOSOne_formatting_sample_title_authors_affiliations.pdf](https://aus01.safelinks.protection.outlook.com/?url=https%3A%2F%2Ftrack.editorialmanager.com%2FCL0%2Fhttps%3A%252F%252Fjournals.plos.org%252Fplosone%252Fs%252Ffile%253Fid%3Dba62%252FPLOSOne_formatting_sample_title_authors_affiliations.pdf%2F1%2F010f0193d41ed7e9-cd185d16-e47c-47ac-add3-a0cc237f3396-000000%2FWwfw3-NuOlws3YP21tPX1wz_dS0lrO-2cRPz6RTPXJs%3D189&data=05|02|amy.mickelberg@research.uwa.edu.au|83dd0e52f0a54645b8a008dd1e86c2dc|05894af0cb2846d8871674cdb46e2226|0|0|638700286661152357|Unknown|TWFpbGZsb3d8eyJFbXB0eU1hcGkiOnRydWUsIlYiOiIwLjAuMDAwMCIsIlAiOiJXaW4zMiIsIkFOIjoiTWFpbCIsIldUIjoyfQ%3D%3D|0|||&sdata=CKUhalEypmTA5DYtoEDwdpi7xu0LCX4wnPI0Cz6a5ME%3D&reserved=0)

The manuscript now meets PLOS ONE’s style requirement.

2. Thank you for stating the following financial disclosure:

 “This research was supported by a Postgraduate Research Scholarship from the Defence Science and Technology Group of the Department of Defence and an Australian Government Research Training Program Scholarship to the first author, an Australian Research Council grant FT190100708 to the third author, and an Office of National Intelligence and Australian Research Council grant NI210100224 to the last author.”

Please state what role the funders took in the study.  If the funders had no role, please state: "The funders had no role in study design, data collection and analysis, decision to publish, or preparation of the manuscript."

If this statement is not correct you must amend it as needed.

Please include this amended Role of Funder statement in your cover letter; we will change the online submission form on your behalf.

This has now been updated and the Role of Funder statement is available to view in the cover letter.

3. Your ethics statement should only appear in the Methods section of your manuscript. If your ethics statement is written in any section besides the Methods, please move it to the Methods section and delete it from any other section. Please ensure that your ethics statement is included in your manuscript, as the ethics statement entered into the online submission form will not be published alongside your manuscript.

Thank you for bringing this to our attention. The ethics statement is now included in the Methods section on p.13 line 298-300.

4. Please include captions for your Supporting Information files at the end of your manuscript, and update any in-text citations to match accordingly. Please see our Supporting Information guidelines for more information: <http://journals.plos.org/plosone/s/supporting-information>.

The supporting information has now been added on p. 39.

We thank the Editor and reviewer for the valuable feedback and suggestions for improvement. As a result, we are confident that the revised work will be a valuable addition to the body of research on the continued influence effect on person impressions.

Best regards

Nicolas Fay
